# A prototype system for the hydrothermal oxidation of feces

**DOI:** 10.1016/j.wroa.2022.100160

**Published:** 2022-11-14

**Authors:** Joël Affolter, Thomas Brunner, Nicola Hagger, Frédéric Vogel

**Affiliations:** a Fachhochschule Nordwestschweiz, Hochschule für Technik, 5210 Windisch, Switzerland; b Paul Scherrer Institut, Laboratory for Bioenergy and Catalysis, 5232 Villigen PSI, Switzerland

**Keywords:** Supercritical water oxidation, SCWO, Hydrothermal oxidation, HTO, Off-grid feces treatment

## Abstract

To ensure access to safe sanitation facilities in rural communities, cheap off-grid technologies need to be developed to substitute pit latrines and open defecation. In this study, we present a prototype system based on hydrothermal oxidation, which, under optimal conditions, converts a fecal sludge simulant almost completely to ${\text{CO}}_{2}$ and water, leaving behind only a carbon-poor aqueous phase with the minerals. The prototype has been designed to process the feces from two households. This technology does not only enable a fast and complete conversion, but is potentially also very energy efficient, as the feed does not require any pre-treatment or drying. The system was found to effectively remove 97–99% of the total organic carbon within a reaction time of 600 s under an external energy demand of roughly 4 kWh per kilogram of wet feces by using the oxygen in air as an oxidant. A total of ten experiments with varying injection pressure, total solids content of the feed, and residence time in the reactor were performed to find experimental settings with high conversion. Only when the residence time was decreased from 600 to 300 s did the conversion fall significantly below 97%. To reach a target value of 99.9% TOC conversion, the reactor temperature and/or the residence time must be increased further. To achieve a system applicable in regions with no connection to the energy grid, the thermal loss of the reactor insulation needs to be lowered further to achieve an overall thermally self-sustaining operation.

## Abstract (author highlights)

• A stand-alone prototype SCWO system to treat fecal sludge was built.
• Carbon conversions over 97% were achieved during reaction times of 600 s.
• The prototype was operated in a cyclic and thermally almost self-sufficient mode.
• An ignition phenomenon was observed and explained theoretically.

## Graphical abstract

**Figure:**
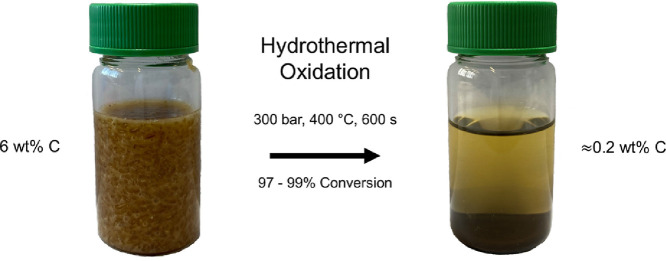

## 1 Introduction

Roughly two billion people still lack access to basic sanitation services. Resulting practices such as open defecation or defecation into latrines, which are seldom or never emptied, can lead to the contamination of drinking water and to the propagation of diseases. While the number of people with access to hygienically safe sanitation facilities is increasing, the progress needs to be accelerated to meet the goal of universal basic sanitation by 2030 (WHO, 2017, WHO, 2021). The missing access to sanitation services is especially pronounced in low-income and remote rural areas. As most of these rural communities also lack access to electricity and sewage systems, self-sufficient off-grid solutions will be key to ensure coverage in such regions. One of the technologies that would allow such an on-site treatment of fecal sludge is hydrothermal oxidation (HTO). Being a high-pressure and high-temperature technology, developing a safe, reliable, and affordable HTO system for rural communities presents an enormous challenge.

Hydrothermal processes take place in aqueous medium at temperatures above 100 °C and at pressures above the corresponding saturation pressure of water. If the conditions exceed the critical point of water at 374 °C and 221 bar, the term “supercritical water process” is also used (Peterson et al., 2008). Under supercritical water conditions, the polarity of water decreases drastically, rendering non-ionic species and gases such as $O_{2}$, $N_{2}$, ${\text{NH}}_{3}$, CO, ${\text{CO}}_{2}$ fully miscible (Brunner, 2009, Brunner, 2009). On the other hand, salts and other minerals are almost insoluble and will precipitate.

In hydrothermal oxidation and supercritical water oxidation (SCWO) an oxidant is added to the water and the organic matter, which can be readily decomposed to form ${\text{CO}}_{2}$, $H_{2}O$, ${\text{NH}}_{3}$, and minerals (Jiang et al., 2020). This has been demonstrated with biological polymers such as proteins and feeds containing polysaccharides (Jin, Kishita, Moriya, Enomoto, 2001, Mochidzuki, Sakoda, Suzuki, 2003, Zhu, Yang, Wang, Jin, Zhong, 2019) as well as their monomers (Holgate et al., 1995), all main components of feces. Human feces are a semi-solid mixture of waste products of human digestion, made up predominantly of water (63–87%) as well as organic and inorganic solids. The main elements in the solid organic fraction are C, O, H, N, and S, found in bacterial biomass, protein, carbohydrates and lipids (Rose et al., 2015).

The treatment of urine/feces waste streams with HTO/SCWO (collectively called HTO herein) was first investigated in relation to life support systems for short or long term manned space missions as well as for lunar bases. In these environments, suitable recycling technologies for human waste are needed due to the high cost of resupplying resources from Earth (Hicks, Hegde, Fisher, 2012, Sedej, 1985). HTO has been identified as an ideal candidate as the reaction allows a fast and complete waste conversion to benign products and can be made self-sufficient. Additionally, the waste stream does not require drying, and nutrients and water can easily be reclaimed (Hicks, Hegde, Fisher, 2012, Sedej, 1985). The first HTO reactors for such purposes were built in 1975–1977 by Lockheed Missiles and Space company (Weitzman, 1977). They reported TOC conversions of 88–94% at 288 to 315 °C and 64 to 150 bar with residence times between 19 and 90 min in a continuous flow reactor using pure oxygen as oxidant. These conditions were still far from supercritical conditions, closer to typical wet oxidation processes. Price (1981) observed conversions in the range of 56–82% at roughly 24 min reaction time, pressures of 157 to 221 bar, and temperatures of 277 to 295 °C. At supercritical conditions, conversions of 88–92% were obtained at shorter reaction times of 2–5 min, pressures of 265–279 bar and temperatures of 400 to 440 °C, but their urine/feces slurry had a higher weight fraction of feces compared to Lockheed.

Takahashi et al. (1989) studied solutions of ammonium hydroxide and acetic acid and a slurry of human urine, feces, and wipes as model wastes for a Controlled-Ecological-Life-Support-System. These model wastes were oxidized with $H_{2}O_{2}$ as the oxidizer in a stirred batch reactor at 250–500 °C at pressures from 47 to 240 bar and residence times up to 120 min. For the mixture of urine, feces, and wipes, temperatures below the critical temperature led to incomplete carbon conversion for a residence time of 60 min. The nitrogen in the ammonium hydroxide was oxidized more slowly. Up to the maximum temperature of 500 °C, less than 50% of the N were oxidized for a residence time of 60 min. Hong et al. (1987) also investigated the treatment of a urine/feces mixture for the same purpose, but focused on supercritical conditions of 256 bar and more than 600 °C. The retrieved product solution contained about 1 ppm of total carbon.

Energy-efficient off-grid technologies to treat human waste are also needed for facilities in remote areas here on Earth. Within the “Reinvent the Toilet Challenge”, Hübner et al. (2016) showed that the organic carbon in fecal sludge can be completely decomposed and oxidized to ${\text{CO}}_{2}$ and CO within 25 min in a batch reactor using air as the oxidant. Miller et al. (2015) investigated the HTO of a dilute simulant feces mixture in a continuous tubular reactor and found greater 99% TOC conversion for a stoichiometric oxygen excess above 20% and reactor temperatures above 535 °C at residence times of ca. 10 s. Within ESA’s MELiSSA project, Zhang et al. (2019) investigated the HTO of a dilute fermenter filtrate in a continuous tubular reactor using $H_{2}O_{2}$ at residence times up to 68 s and temperatures between 300 and 380 °C. At these conditions, the TOC removal efficiency was always below 70%.

In this study, the conversion of a fecal sludge model mixture was investigated under different experimental settings using a semi-automated prototype HTO reactor with ambient air as the oxidant. The goal was to study the HTO of fecal sludge one step closer to an automated, full-scale system running in a thermally self-sufficient mode. Eventually, such a system would have to fulfill the private standard for reinvented toilets (International Organization for Standardization, 2020). Here, the focus was set on the conversion of the injected synthetic feces feed (SFF) and the energy consumption. The carbon conversion and the energy consumption were measured depending on the injection pressure, the reaction time as well as the total solids (TS) content of the feed. Nitrogen is another important element, which was not monitored in this study and is recommended to be the subject of future work.

## 2 Rationale

Drawing from the experience reported in the literature about fecal sludge management and designing and operating HTO systems, several key learnings were derived for the design of the prototype system, such as:

• Fecal sludges can have a broad range of solids concentrations. Preprocessing the sludges to a constant dry solids content is a challenge on its own. A robust HTO system will be able to process a wide range of solids concentrations.
• Urine-separated feces should be preferred, as they have the advantage of lower chloride content and thus a lower potential for corrosion in HTO systems.
• The mineral fraction produced in HTO must be recovered for reuse as fertilizer.
• Air is by far the cheapest, most abundant, and safest oxidant available.
• Continuous HTO systems have traditionally been preferred over batch systems for their higher efficiency. While such continuous systems can use direct heat recovery via heat exchangers, batch systems need a storage capability to transfer heat from one batch to the next.
• Pumping heterogeneous sludges containing particles at low flow rates is a big challenge.
• The batch-wise operation of a household toilet calls also for a batch-wise treatment of the feces to avoid large storage tanks, which would entail additional challenges such as anaerobic decomposition of the sludge and odor emissions.
• Thermal inertia of batch systems is high. This bears potential for heat recovery by solid heat storage materials.

Continuous HTO systems seem advantageous for larger scale, multi-user toilets, while a batch system appears as the most appropriate choice for small-scale, household systems.

The main unit operations required in an HTO system are: i) preprocessing, ii) feeding, iii) heating and reacting, iv) discharging. For a system to be thermally efficient, heat recovery is an additional necessary unit operation.

### 2.1 Preprocessing

Preprocessing of the fecal sludge is a very important step to enable a smooth and sustained operation of the feces treatment unit for a broad range of feed compositions. Initially, a wet grinding device (InSinkerator) was installed just below the feed container. It was later replaced by an in-line macerating pump (TA3P10-19/24V, Johnson) that allowed a more reproducible filling of the feed injector. This macerator cuts down larger, soft and fibrous particles and homogenizes the solid mixture. It is not able to process large, hard particles and tough parts such as plastic foil.

### 2.2 Feeding

Feeding a viscous sludge containing particles of different size, shape, and hardness, and whose consistency may vary largely, poses an enormous challenge to any feeding system for high pressure operation. One requirement for a robust toilet was its ability to cope with all the intake that the users would produce. It may well be that items other than feces would end up in the toilet bowl and finally in the inlet to the treatment unit. One important decision when designing the prototype FOX-02 was to avoid any kind of mechanical pump for the feces on the high pressure side. The concept of FOX-02 uses air both as the propellant for the feces and as the oxidant, avoiding any moving part (except for some valves) for the feeding under high pressure.

### 2.3 Heating and reacting

High destruction efficiencies of organic carbon require a good control of the reaction parameters, i.e. local availability of the oxidant via a well-mixed reacting fluid, and both temperature and residence time high enough to oxidize also recalcitrant compounds. For supercritical reaction conditions, a complete miscibility of the gaseous oxygen (and nitrogen if the oxidant is air) and the fluid phase is expected (Peterson et al., 2008). The solid phase may pose a bigger challenge. Before the organic solids can oxidize, they need to be liquefied and mixed with the oxidant on a molecular scale. Liquefaction is typically a slower process than oxidation (Peterson et al., 2008), adding to the total time needed for reaction. In the FOX-02 setup, the rapid expansion of the air/sludge mixture under high pressure into the hot reaction chamber at low pressure presumably produces a highly turbulent spray of small droplets and particles, surrounded by air, ensuring a high mixing rate as well as a high heating rate. Heat-up rates of 2 to 6 K/s from 290 to 390${}^{\circ }$C have been achieved for the feed mixture in that setup.

When biomass is heated up slowly under hydrothermal conditions, some of its constituents may undergo dehydration reactions, leading to coke formation, before being oxidized. Coke itself is hard to oxidize (Matsumura et al., 2002) or gasify in supercritical water (Matsumura et al., 1997). Thus another rationale for heating up the fecal sludge quickly is the avoidance of coke formation by rapidly passing through the window of temperatures promoting coke formation. Under non-oxidizing conditions, Müller found this temperature window to be around 350–370${}^{\circ }$C for small organic compounds such as glycerol (Müller and Vogel, 2012), but solid biomass particles may carbonize already at lower temperatures. Coke formation is also possible under oxidizing conditions, especially at low oxygen-to-carbon ratios and when the mixing of oxygen, water, and organics is slow. Hübner et al. found some coke and tar formation in their batch experiments with fecal sludge at low oxygen-to-carbon ratios (Hübner et al., 2016). Another situation leading to coke formation is the disappearance of the liquid phase when crossing the vapor region. To evaluate the probability of coke formation by this pathway in the FOX-02 setup, the p-T trajectories, as depicted in Fig. 2c, were recorded.

### 2.4 Discharging

In the FOX-02 concept, a rapid discharge of the hot reactor contents is achieved by suddenly opening the exit valve and allowing the mixture to flow into a vessel of larger volume than the reactor. This rapid discharge has several aims:

• quenching the reaction by reducing the temperature of the mixture very rapidly
• flushing particles out of the reactor vessel by entraining them with the fluid at a high velocity to avoid settling of particles
• emptying the reactor vessel while keeping it hot, i.e. no active cooling of the vessel before discharge

Due to the high pressure difference during discharge a critical (or choked) flow is established across the exit valve. Such a high velocity flow creates challenges for the valve. Hard mineral particles may lead to erosion and abrasion of the needle and/or the seat. Also, the large temperature difference between the fluid and the valve body of around 200 K induces thermal stress on this component. The discharge valve V7 was found to leak after a random number of cycles, making it the most critical part of the current design.

### 2.5 Heat recovery

The approach chosen for the FOX-02 prototype is based on two half-shells made of low-grade steel, attached firmly to the external surface of the HTO reactor vessel. The half-shells allow for easy handling during assembly and disassembly. Heat would be transferred from the hot reactor wall to the storage shells by conduction, and vice versa, depending on the sign of the temperature gradient between the reactor and the shell. The shells have been fitted with electric heating cartridges to provide the energy for the initial heating-up of the reactor. A safety thermocouple, connected to a safety shut-off controller, prevents the shells, and consequently the reactor, from overheating. For this system to work efficiently, a high performance thermal insulation was used around the reactor and the heat storage shells. Heat recovery from the exothermic reaction and its storage for several hours is an important feature for such a stand-alone batch system, as the toilet will experience intermittent operation and longer idle times. The heat storage capacity of the FOX-02 setup was neither optimized nor designed to meet a specific target. It was a first best-guess design to show in principle the performance of such a system. As a next step, alternative heat storage materials may be selected and tested for a longer heat storage capability.

## 3 Materials and methods

### 3.1 FOX-02 setup and operation

**Fig. 1:**
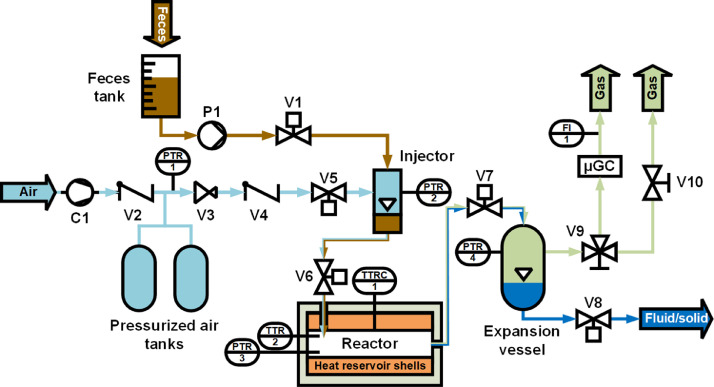
**Simplified scheme of the FOX-02 setup.** The flow of SFF and air are marked brown and light blue, respectively. The mixture ejected from the reactor contains water, minerals, oxidation products, and gases, which is separated into gas (green) and liquid/solids (blue) in the expansion vessel. The size of the individual components is not drawn to scale. (For interpretation of the references to colour in this figure legend, the reader is referred to the web version of this article.)

FOX-02 is a prototype for conducting repeated HTO batch tests in an automated manner. A simplified scheme of the FOX-02 setup is displayed in Fig. 1 and a picture of the setup is shown in the supplementary Fig. S1.1. A list of components is given in the supplementary Table S1.1. The feed is stored in a tube on top of which a graduated 1000 mL cylinder has been mounted to determine the injected volume. At the beginning of a cycle, the feed is pumped into the injector at ambient pressure by turning on the macerating pump P1 and opening the ball valve V1 for five seconds. Then, the injector is pressurized with air by opening valve V5. Valve V6 is then opened, leading to the injection of the SFF-air mixture into the preheated reactor. Two seconds later, V5 and V6 are closed again. Roughly, 35–50 mL of SFF are injected per cycle in this way. The feed mixture reacts under hydrothermal conditions at around 250 to 320 bar for the set residence time, after which the needle valve V7 is opened, ejecting the hot mixture into the expansion vessel (internal volume 3 L). The water vapor condenses in the colder expansion vessel. The expansion vessel heats up to about 30–70 °C after each emptying cycle, and it cools down again between cycles by losses to the environment. The permanent gases, saturated with water vapor, are ejected from the expansion vessel at the top via the valves V9 and V10. To analyze the amount and the composition of the flue gas in every fifth cycle, V9 is switched towards the μGC and a gas meter FI1. After the reacted fluid has been ejected, the injector is still pressurized and might contain some residual feed. The injector content can therefore not simply be released into the expansion vessel and instead is injected with air into the reactor after closing V7 again. The content is kept in the hot reactor, but at low pressure, for 20 s to allow the oxidation of the residues. Then, the reactor content is once again released via the expansion vessel. The residual pressure from the secondary reaction is used to drain the process water from the expansion vessel via valve V8. The process water is collected in a tank and analyzed only after the whole series of cycles has been completed.

The amount of SFF injected was determined from the level drop in the graduated feed cylinder over the course of a measurement series. For the evaluation, the volume of feed injected was averaged over all cycles. Additionally, the amount of SFF injected was determined by the weight difference of the feed storage canister after filling up to the starting level once all cycles were completed. The amount of product solution ejected from the reactor was determined from the weight difference of the collection vessels before and after all cycles were completed. The volume and composition of the gas ejected from the reactor were measured after every fifth cycle. The pressurized air used for the reaction and for switching the pneumatic valves is generated by a compressor and stored in two 6 L pressurized air tanks. Pressure sensors are connected to the pressurized air vessels (PTR 1), injector (PTR 2), reactor (PTR 3) and to the expansion vessel (PTR 4). The temperature inside the reactor is measured by the thermocouple TTR 2. Heating of the reactor vessel is controlled with TTRC 1, which is placed in a groove between the heating shells and the reactor vessel. The reactor is of cylindrical shape with an inner diameter of 60 mm, has an inner volume of 1000 mL and is made of Alloy 286 (No. 1.4980). Two half-shells made of low-grade steel (No. 1.0045, 36.4 kg each shell) are placed around the reactor in direct contact with the vessel. One heating cartridge is placed in each half-shell and is connected to a temperature controller. The reactor is equipped with a rupture disc (400±40 bar) and can operate at a maximum temperature of 500 °C. Its use is limited to 20,000 cycles. This value was chosen for the design of the reactor in accordance with the code AD2000. The pressure vessel is checked regularly by SVTI, the Swiss inspectorate for pressure vessels. The reactor is insulated by a ceramic fiber insulation, which is between 80 to 150 mm thick. The operation of the FOX-02 is controlled by a PLC and a custom-made control and data acquisition software.

### 3.2 Synthetic fecal sludge

Not only is it difficult to source large amounts of fecal sludge with a high total solids (TS) content, but different batches of fecal sludge typically exhibit a large variability in composition, heating value, and chemical oxygen demand. For this systematic study, a synthetic feces feed (SFF) mixture proposed by Penn et al. (2018) was slightly adapted and used instead (see SI for exact composition). A batch of roughly 3.5 kg per experiment was prepared by adding all components and the desired volume of water in a bucket on the day before the experiment. The mixture was stirred thoroughly and kept below 10 °C overnight. The chemical composition and physical properties of the solid part of SFF were determined as described in Section 3.4 and can be found in Table 1.

**Table 1:** **Chemical and physical properties of SFF.** Measured properties of dry SFF (this study), and range of values for human feces from the literature. ^a^ based on dry mass. ^b^ O = 100 - (wt% of C+H+N+S+ash).

	dry SFF	Human Feces	References
HHV [MJ /kg]^a^	22.6 ± 0.6	6 - 45	Rose et al. (2015), Onabanjo et al. (2016a), Monhol and Martins (2015)
COD [g / gTS]	0.96 ± 0.06	0.57 - 1.67	Rose et al. (2015)
C [wt%]	49.8 ± 0.6	44 - 55	Rose et al. (2015)
H [wt%]	7.22 ± 0.03	7 - 7.6	Onabanjo et al. (2016b), Onabanjo et al. (2016a), Monhol and Martins (2015)
N [wt%]	2.51 ± 0.06	5 - 7	Rose et al. (2015)
S [wt%]	0.3 ± 0.2	0.87 - 1.6	Rose et al. (2015), Monhol and Martins (2015)
Ash [wt%]	7.3 ± 0.6	9.7 - 14.6	Monhol and Martins (2015), Onabanjo, Kolios, Patchigolla, Wagland, Fidalgo, Jurado, Hanak, Manovic, Parker, McAdam, Williams, Tyrrel, Cartmell, 2016, Onabanjo, Patchigolla, Wagland, Fidalgo, Kolios, McAdam, Parker, Williams, Tyrrel, Cartmell, 2016
O [wt%]	33 ± 2^b^	32.1	Monhol and Martins (2015)

### 3.3 Experimental design

The goal of this experimental series was to determine reaction parameters that allow a high conversion under low external energy demand. In this setup, only the reactor preheat temperature, the injection pressure, the TS content of the SFF, and the reaction time can be set by the operator and are thus considered reliable experimental variables. Other variables such as the amount of SFF injected or the oxygen-to-fuel equivalence ratio can only be determined indirectly and are thus not experimental variables. The experimentally accessible range for each variable is limited by safety and technical considerations, which will be shortly summarized here.

The residence time $t_{residence}$ was set to either 300 or 600 s to investigate its effect on the total conversion and on the energy demand. Since the goal was to achieve high carbon conversions, shorter reaction times were not explored. The injection pressure $p_{injection}$ was varied between 160 and 120 bar, allowing us to lower the oxygen-to-fuel equivalence ratio and limit the amount of air injected per cycle while still reaching supercritical conditions. The TS content of the SFF was varied between 9 and 15 wt% to simulate the varying thickness of the stool entering the reactor. Under these conditions, reactor pressures between 250 to 320 bar were reached. Some combinations, such as high injection pressures and high TS contents or low injection pressure and low TS contents were not tested. While the former conditions might lead to bursting of the rupture disk due to pressure reaching the limit of 400±40 bar, the latter could lead to low reactor pressures and subcritical conditions, where coke formation occurs. In addition, the SFF separated into its oily and watery phases at TS contents below ca. 10 wt%, which led to a low reproducibility of the injected amount of carbon (see Test11 and Test12 in Table 2).

**Table 2:** Overview of the experimental results. Experimental settings (columns 1–4) and experimentally determined parameters of all series (columns 5–12). Uncertainties correspond to one standard deviation calculated via Gaussian error propagation. Test06, Test07 and Test08 had to be stopped without analyzing the product gas or liquid due to leakages or COVID related issues and are therefore not displayed here. n/a: not available. All Tests consist of 15 cycles. ^a^ In several series, the ignition peak was only present in some cycles. These are marked with yes*. ^b^ The heat generated by the complete oxidation of the SFF is roughly 0.8 kWh/kg, assuming a typical TS of 12.5 wt% and using the HHV value from Table 1. ^c^ Test09 is a repetition of Test05, which showed leakages in the last three cycles and is thus not further discussed. ^d^ In series Test11 and Test12, separation of the feed was observed, giving misleading λ, $X_{TOC}$, $X_{{\text{CO}}_{2}}$ and carbon balance values as the amount of carbon injected is overestimated.

Series	$t_{residence}$	$p_{injection}$	$TS_{Feed}$	${\text{Ignition}\;\text{Peak?}}^{a}$	$V_{Feed}$	$m_{Product}$	${E_{heater}}^{b}$	λ	$X_{TOC}$	$X_{CO_{2}}$	C-Balance
	[s]	[bar]	[wt%]		[mL]	[g]	[kWh/kg]	[–]	[–]	[–]	[–]
Test01	600	160	15.2±0.6	yes	37±1	28	1.6±0.2	2.3±0.2	0.99±0.01	0.95±0.06	0.97±0.05
Test02	600	160	11.2±0.2	no	46±1	41	1.3±0.2	2.3±0.2	0.98±0.01	0.93±0.06	0.97±0.05
Test03	600	140	12.9±0.7	yes*	46±1	41	1.2±0.2	1.9±0.2	0.99±0.01	0.87±0.07	0.89±0.04
Test04	600	140	15.2±0.7	yes	37±1	36	1.4±0.1	2.0±0.3	0.99±0.01	1.03±0.09	1.05±0.07
Test05^c^	300	140	15.4±0.5	yes	43±1	40	n/a	1.9±0.2	0.97±0.04	0.82±0.07	0.85±0.06
Test09	300	140	15.1±0.2	yes	41±1	42	0.7±0.1	1.7±0.2	0.87±0.01	0.84±0.07	0.98±0.06
Test10	300	160	15.1±0.2	yes	42±1	39	n/a	1.9±0.2	0.92±0.01	0.86±0.07	0.94±0.06
Test11^d^	600	160	9.4±0.2	no	50±1	46	1.9±0.2	2.6±0.3	0.94±0.05	0.62±0.05	0.70±0.05
Test12^d^	600	140	9.4±0.2	no	54±1	46	1.5±0.2	2.1±0.3	0.86±0.03	0.66±0.05	0.84±0.05
Test13	600	180	11.3±0.1	yes*	46±1	45	1.2±0.2	2.5±0.3	0.97±0.01	1.08±0.08	1.12±0.08
Test14	300	160	11.0±0.2	yes*	49±1	44	1.1±0.1	2.2±0.3	0.91±0.01	0.90±0.07	1.00±0.07

For each set of parameters investigated during the campaign, 15 cycles were performed. One cycle corresponds to one batch test. It starts with (re)heating the reactor and filling a feed sample into the injector. The cycle ends with the release of the reacted mixture from the reactor. Before each set of 15 cycles ten start-up cycles were necessary to reach reproducible injections. These start-up cycles were disregarded for the analysis as the conditions still changed drastically in some cases. The process water was collected in the same vessel for all 15 cycles, producing an averaged sample for each set of 15 cycles. The amount of gas ejected and the gas composition were measured after every fifth cycle.

### 3.4 Analytics

The TS content of the SFF and of the product solution were determined by drying the wet sample at 105 °C in a Moisture Analyzer (Mettler Toledo HE53). The ash content was determined in accordance with the standard DIN EN 14775 by heating the dry sample to 250 °C in 0.5 h, holding the temperature for 1 h, heating to 550 °C in 0.5 h in air and holding the final temperature for 1.5 h. The chemical oxygen demand (COD) was determined using the NANOCOLOR® CSB LR 150 test (Macherey-Nagel). The CHNS composition was analyzed using a VarioElcube (Elementar, Hanau, Germany). The oxygen fraction in the dried feed was calculated by subtraction of the weight percentages of C, H, N, S, and ash from 100%. The HHV was determined with an IKA C1 bomb calorimeter (Cole Parmer, Illinois, USA). To determine the TC/TIC/TOC content of the product solution, the solution was diluted to lie within the experimentally accessible range of 10–1000 ppm TOC/TIC/TC and measured with a DIMATOC 200® (DIMATEC, Essen, Germany).

### 3.5 Evaluation of results

#### 3.5.1 Oxygen-to-fuel equivalence ratio λ

The oxygen-to-fuel equivalence ratio λ is defined as the amount of oxygen present in the system at the beginning, $n_{O_{2},0}$, divided by the stoichiometric oxygen demand of the feed, $n_{O_{2},stoich}$. This ratio can be written as

(1) $$\lambda =\frac{n_{O_{2},0}}{n_{O_{2},stoich}}=\frac{n_{O_{2},0}}{\xi_{feed,dry}m_{feed,dry}}$$

with the amount of dry feed $m_{feed,dry}$ injected per cycle and the chemical oxygen demand per mass $\xi_{feed,dry}$. The amount of oxygen $n_{O_{2},0}$ present in the reactor at the beginning was calculated by the pressure drop in the gas cylinder during injection using the real gas equation with the van der Waals coefficients (Atkins and Paula, 2019) $a=135.8\cdot 10^{-3}\text{Pa}\;{\text{m}}^{6}\;{\text{mol}}^{-2}$ and $b=0.0364\cdot 10^{-3}\;m^{3}\;{\text{mol}}^{-1}$ and the content of oxygen in ambient air, $y_{O_{2}}=0.21$ mol/mol.

#### 3.5.2 Carbon conversion

The conversion of the SFF was calculated by two independent ways, i.e., as $X_{TOC}$ from the amount of total organic carbon (TOC) left in the product solution and as $X_{{\text{CO}}_{2}}$ from the amount of ${\text{CO}}_{2}$ formed with

(2) $$X_{TOC}=1-\frac{n_{TOC_{out}}}{n_{TOC_{0}}}$$

(3) $$X_{{\text{CO}}_{2}}=\frac{n_{{\text{CO}}_{2}}}{n_{TOC_{0}}}$$

where $n_{TOC_{out}}$ is the amount of TOC in the product solution, $n_{TOC_{0}}$ the amount of TOC injected into the reactor and $n_{{\text{CO}}_{2}}$ the amount of ${\text{CO}}_{2}$ generated per cycle. Carbon-containing residues such as coke were not found and therefore not included in the evaluation. The amount of TOC injected per cycle was calculated using the TOC content of the dry feed $\omega_{TOC,feed,dry}$, the TS content of the feed $\omega_{TS,feed}$, the volume of feed $V_{feed}$ injected per cycle, the density of the feed $\rho_{feed}$ and the molar mass of carbon $M_{C}$. The density of the feed in the used TS range was found to be 1 g/mL.

(4) $$n_{TOC_{0}}=\frac{\omega_{TOC,feed,dry}\cdot \omega_{TS,feed}\cdot V_{feed}\cdot \rho_{feed}}{M_{C}}$$

The amount of TOC in the product solution was calculated from the TOC concentration in the product solution $c_{TOC,out}$ and the volume of product solution ejected $V_{ps}$.

(5) $$n_{TOC_{out}}=\frac{c_{TOC,out}V_{ps}}{M_{C}}$$

The amount of ${\text{CO}}_{2}$ generated was calculated using the volumes of gas injected $V_{air,0}$ and ejected $V_{gas,out}$ at standard conditions, the molar ratio of ${\text{CO}}_{2}$ in the injected $y_{{\text{CO}}_{2},air}$ and ejected $y_{{\text{CO}}_{2},out}$ gas and the molar volume of the ideal gas $V_{m}$.

(6) $$n_{{\text{CO}}_{2}}=\frac{V_{gas,out}\cdot y_{{\text{CO}}_{2},out}-V_{air,0}\cdot y_{{\text{CO}}_{2},air}}{V_{m}}$$

#### 3.5.3 Carbon balance

The carbon balance was calculated as the ratio of the molar amount of all ejected carbon, $n_{C,out}$, to the molar amount of all injected carbon, $n_{C,in}$. Because the feed contained nearly no inorganic carbon, the amount of carbon injected is equal to the molar amount of TOC, $n_{TOC_{0}}$. Ejected carbon was accounted for in the gas phase as $n_{{\text{CO}}_{2}}$ and in the product solution as $n_{TC_{out}}$. While our GC was able to detect also CO and ${\text{CH}}_{4}$, the concentration of theses compounds was around the quantification limit of the instrument and their contribution was thus not included.

(7) $$CB=\frac{n_{C,out}}{n_{C,in}}=\frac{n_{{\text{CO}}_{2}}+n_{TC_{out}}}{n_{TOC_{0}}}$$

## 4 Results and discussion

The experimental parameters as well as the energy demand of the heating shells $E_{heater}$, the oxygen-to-fuel equivalence ratio λ, the conversions $X_{TOC}$ and $X_{CO_{2}}$, and the carbon balance of all tests are displayed in Table 2. More information regarding the TOC content of the process water and the gas composition of the off-gas can be found in the Supplemental Information Table S3.3.

### 4.1 Carbon conversion and carbon balance

In all experiments, the SFF was converted to a semi-transparent suspension containing only a fraction of the initial carbon. The injected feed contained 75 000 mg/L of TOC at a TS of 15 wt%, which was transformed into ${\text{CO}}_{2}$ as the main gaseous product, and dissolved organic carbon (DOC), together with carbonates, under all experimental conditions. The TOC concentration of the final process water was between 455 and 9000 mg/L, the ejected gas contained between 5–9 mol% of ${\text{CO}}_{2}$ (see Table S3.3). The overall carbon conversion was in the range of 97%. Such high conversion values lead to the assumption that no significant inhomogeneities or dead zones were present in the reactor. The faint color of the product solution, compared to similar work of Miller et al. (2015), most probably stemmed from the higher TS concentrations and lower peak temperatures used in our study. Only traces of CO and ${\text{CH}}_{4}$ were found in the gas phase. This is remarkable, as in earlier batch experiments with real feces, Hübner found some of the carbon being converted to CO (Hübner et al., 2016). Test09 is the experiment most likely to produce CO because it has the lowest oxygen-to-fuel ratio, a short reaction time, and a high feed concentration. The concentration of CO in the gas from Test09 was below 0.3%, which was just around the quantification limit. The biggest difference between the batch reactor and the FOX-02 setup is the rapid heating up and mixing of oxygen, water, and organics in the FOX-02 during the injection, as opposed to the static situation in the batch reactor used by Hübner. In the latter case, it was found that most of the carbon conversion happened already during the subcritical heating up, during which still a liquid phase prevailed. A fraction of the initial carbon was transformed to CO, most likely by decarbonylation of partially oxidized organics. The oxygen had first to dissolve in the liquid phase and diffuse to the organics and to the dissolved CO to oxidize it to ${\text{CO}}_{2}$. Our hypothesis is that in the FOX-02 supercritical, monophasic conditions are reached much more rapidly than in Hübner’s batch reactor, accelerating the oxidation of the CO formed from the decarbonylation of the organics.

**Fig. 2:**
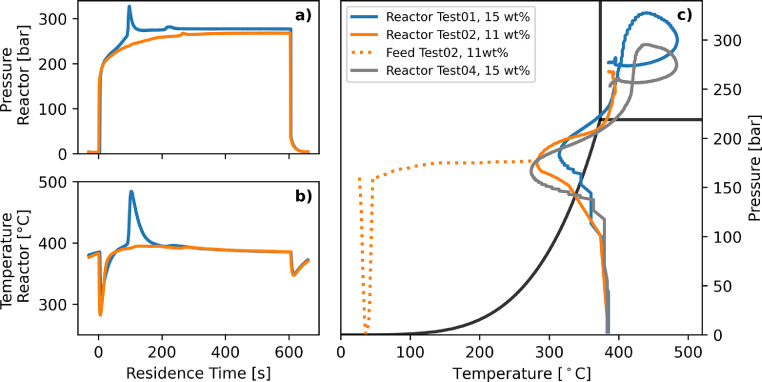
**Pressure and temperature profile.** a) Pressure and b) temperature profile of the reactor during a cycle from Test01 (blue) with a TS content of 15 wt% exhibiting an ignition peak and a cycle from Test02 (orange) with a TS content of 11 wt% without noticeable ignition peak. c) p,T trajectories of the reactor during the two cycles from a) and b) from t=0 to 600 s as well as the speculated trajectory of the feed (dashed, orange). The trajectory of a selected cycle from Test04, where the highest CO formation is expected, is shown in gray. The phase boundary of water is displayed in black. (For interpretation of the references to colour in this figure legend, the reader is referred to the web version of this article.)

In earlier experiments with real human feces so-called “ignition peaks” in temperature and pressure were observed to occur, if the TS content of the feed exceeded 12 wt%. The SFF used in the present study showed a similar behavior. The typical pressure and temperature profile measured inside the reactor during two selected runs with a TS content (a) above and (b) below this critical TS content of about 12 wt% is shown in Fig. 2. The pressure and temperature profiles of all series are depicted in the supplementary Figs. S3.1 and S3.2.

Upon injection of the feed and the pressurized air from the injector at t=0, the pressure in the reactor increased sharply, irrespective of the TS content. Simultaneously, the temperature dropped by about 100 °C due to the injection of the cold feed. As the feed heated up, the temperature returned to the set-point temperature of 390 °C, while the pressure increased further due to the heat up of the injected mixture. If the TS content of the feed was sufficiently high (15 wt% in this case, Test01, blue), both pressure and temperature increased rapidly after about 2 min, denoted as ignition peak. During this ignition peak, the maximum pressure and temperature reached about 330 bar and 485 °C, respectively. Due to the symmetrical design of the reactor we assume that the temperature peak occurred throughout the whole reactor volume. However, every thermocouple has an inherent response inertia and may not be able to detect temperature peaks on very short time scales. In some cases, a second, less distinct ignition peak (Δp about 10 bar and ΔT about 5 °C) was observed. If feed with a TS content lower than 11 wt% was injected at an identical preheating temperature and injection pressure, no such ignition peaks were visible (compare Test01 with Test02 in Fig. 2). The occurrence of ignition peaks does not seem to affect the conversion, even though their higher temperatures would lead to faster reaction rates. Hübner concluded that only a relatively small fraction of volatile organics were responsible for the ignition peaks, and this small fraction would thus not influence the overall carbon conversion much (Hübner et al., 2016). Comparing the experiments in this study with and without ignition peaks, the same conclusion can be drawn, as the conversion is not higher for the experiments exhibiting ignition peaks. Similarly, no relation between the occurrence of ignition peaks and the CO concentration was found.

The resulting pressure-temperature (p-T) trajectories of the high and low TS case are displayed in Fig. 2c in blue and orange, respectively. The hypothetical p-T trajectory of the feed itself follows a different initial course (dotted orange line), as the feed is already pressurized in the injector prior to the injection, but is not at the reaction temperature yet. Once the valve V6 is opened, the pressure inside the injector drops while the feed is injected into the reactor where it is quickly repressurized. The feed now heats up rapidly to reactor temperature, indicated by the dotted line merging after about 3 s with the p-T trajectory derived from the temperature sensor inside the reactor. We found that the time after which supercritical conditions are reached strongly correlates with the injection pressure. At high injection pressures of 160 or 180 bar, it takes between 25 to 40 s to reach supercritical conditions after injection, while it takes between 60 to 90 s at an injection pressure of 140 bar. This means that depending on the residence time and injection pressure, the feed spends around 70 to 95% of the total reaction time under supercritical conditions. This trajectory is important to detect whether the feed mixture passes through the vapor phase region or whether it always stays liquid up to the transition to the supercritical region.

A reduction of the injection pressure from 160 to 140 bar resulted in a decrease of the oxygen-to-fuel ratio λ, which did not have an effect on the conversion (compare Test01 and Test 02 with Test04 and Test03, respectively). From an energetic and economic standpoint, it is preferable to inject the feed with as little pressure as possible, as long as λ>1 and supercritical conditions are reached in the reactor, because this minimizes the operating time of the compressor. However, in previous experiments with a small batch reactor, the formation of coke and tar was observed, when the p-T trajectory crossed the vapor-liquid equilibrium line of water (Hübner et al., 2016). Coke and tar form under oxygen-deficient conditions (i.e., when λ<1) and only slowly decompose once formed. Therefore, coke and tar formation must be prevented, as it will ultimately lower the conversion, contaminate the liquid product phase, and may lead to clogging of valves and pipes over time. Passing through the vapor region will “dry out” the liquid phase, resulting in very high concentrations in the remaining liquid, which promotes coke formation. A trajectory from Test04, with the same TS content as in Test01 (blue) but with an injection pressure of only 140 bar instead of 160 bar, is displayed in Fig. 2c in gray. Because of the lower injection pressure, the vapor pressure curve was crossed and coke formation may have taken place. However, it was not possible to determine the amount of coke in the effluent from these tests.

The only series in which $X_{TOC}$ was below 97% were experiments run with a shorter residence time of 300 instead of 600 s. This indicates that a residence time of more than 300 s is needed to achieve (almost) complete conversion in the current setup. The low conversion in Test11 and Test12 is due to a separation of the feed into its oily and watery phases in the feces tank. Because of the phase separation, the feed becomes inhomogeneous and the amount of carbon injected per cycle is subject to a large uncertainty. The carbon conversion $X_{CO_{2}}$ and the C-balance of these two experiments were low. Probably, the phase separation resulted in less carbon-rich feed being transferred into the injector. We therefore excluded these two series from the further discussions.

The mass of the ejected product solution was consistently about 20% lower than the mass of the injected feed. This difference in total mass can be explained by i) the decomposition of the solid feed fraction into gases and ii) water losses by saturation of the ejected gas stream with water vapor.

### 4.2 Energy balance

**Fig. 3:**
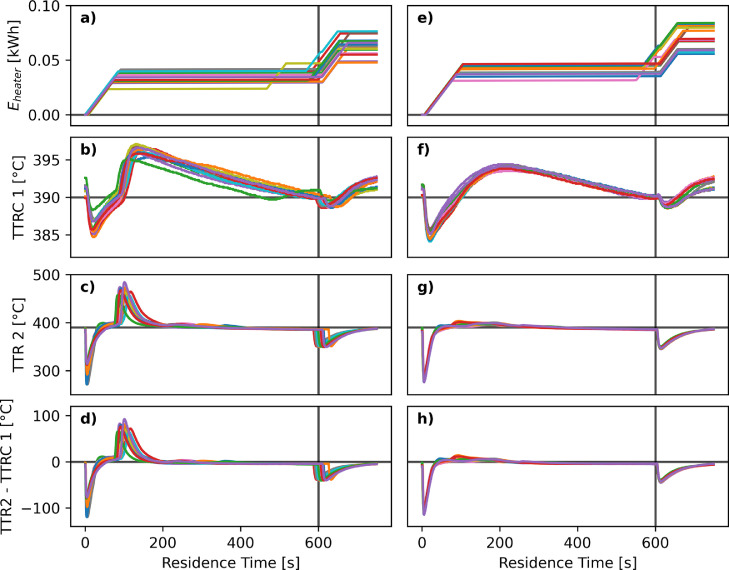
**Energy demand of the heating shells.** The left column shows the cumulative energy demand of the heating shells (a) from the feed injection at t=0 s, the TTRC 1 (b) and TTR 2 (c) temperatures as well as the difference between TTR 2 and TTRC 1 (d) for all cycles from experiment Test01 with a high TS content. The right column displays the same graphs e, f, g, and h for all cycles from experiment Test02 with a lower TS content. The vertical line at t=600 s indicates the opening of valve V7.

To make the feces treatment unit applicable in remote regions, the energy demand needs to be as low as possible, ideally to be covered by a small array of solar panels on top of the toilet. The two most energetically demanding process steps in the FOX-02 are the heating of the reactor and the compression of the air. Fig. 3a and b show the cumulative energy demand of the heating shells starting from the injection for Test01 and Test02 with a TS content of 15 and 11 wt%, respectively.

In both cases, about 0.05 kWh was needed per cycle to keep the temperature of the shells at 390 °C. The heating switched on as soon as the temperature sensor TTRC 1, located in the groove between the heating shells and the outer surface of the reactor, dropped below 390 °C (Fig. 3b and f). This happened in the beginning after the injection of the cold feed, and at the end after the ejection of the hot fluid. FOX-02 did not require any external heating during the reaction time, which was due to the heat generation from the exothermic feed conversion in the insulated reactor vessel. Over all series, the system required between 0.03 and 0.05 kWh of heating energy per cycle, which equals to 0.7 to 1.6 kWh per kilogram of wet feed (37 to 49 g per cycle). In comparison, the heat provided by complete oxidation of SFF with 12.5 wt% TS released 0.8 kWh per kilogram of wet feed, which is in the same range as the energy provided by the heaters.

From a comparison of the temperature at the outer surface of the reactor (TTRC 1, Fig. 3b and f) and the temperature inside the reactor (TTR 2, Fig. 3c and g), the effect of the large thermal mass of the reactor and the shell can be seen. While the temperature variation at the heating shell is about $\pm 5^{\circ }$C, the temperature inside the reactor can increase or decrease by almost $\pm 100^{\circ }$C. The slow decrease of TTRC 1 from the peak value to the ejection point of the reactor contents is due to the heat losses to the surroundings. In Fig. 3d and h the difference TTR 2–TTRC 1 has been plotted to highlight the sign of the heat flux between the reactor contents and the shell. When it is positive, heat flows from the fluid inside the reactor to the reactor wall and the shell. When it is negative, heat from the shell is transferred to the reactor and the fluid. The energy demand of the FOX-02 system may be reduced further by using better insulation material and also by allowing less gaps around tubing, sensors, etc. In principle, the average temperature of the reactor and the shells should slightly increase with each cycle, due to the exothermic heat of reaction. Because the insulation is not perfect, the temperatures TTR 2 and TTRC 1 both do not increase with each cycle, as seen in Fig. 3.

The compressor requires about 0.12 kWh per cycle or 3 kWh per kilogram of wet feed to replenish the air ejected from the gas cylinder in the previous cycle. Because the control circuit and the pump require very little energy in comparison, the overall energy consumption of the system is on average about 4 kWh per kilogram of wet feed injected (3 kWh from the compressor, 1 kWh from the heating shells). Additionally, the gas tanks were refilled after every injection to improve the reproducibility of the injected amount, which led to a high energy demand as the pressure repeatedly needed to be built up to 280 bar before the gas tanks could be filled. For a real-world application, the air tanks filled to 310 bar contain enough air to allow 10 to 12 injections with 160 bar.

### 4.3 Transient thermal behavior

The reaction system realized in the FOX-02 setup corresponds to an autothermal oxidation with heat transfer to the storage shells. The heat liberated by the exothermic oxidation reactions increases the temperature of the reacting mixture, accelerating the oxidation kinetics. This leads to a faster heat release rate, which corresponds to a self-acceleration, also known as “runaway reaction” in safety considerations. This system can be described and analyzed by a transient heat balance, when the kinetics are known. A simplified and generalized theory of such systems was proposed by Semenoff (1928). In this work, Semenov’s theory was applied to describe the ignition peaks. For such a peak to occur, the heat release rate must be significantly faster than the heat transfer rate to the reactor wall. In the opposite case, where the heat transfer rate to the wall is of similar or higher magnitude than the heat release rate, only a small peak or no peak at all is assumed to occur.

Semenov’s theory assumes a batch reactor with irreversible first order kinetics. Also a constant wall temperature and no consumption of the reactant is assumed. Even if some of these assumptions are violated, Semenov’s theory is a good approximation for various real systems (Varma et al., 1999). The kinetics of Hübner et al. (2016) for hydrothermal fecal sludge oxidation and direct irreversible decomposition of TOC to ${\text{CO}}_{2}$ with a single activation energy were used to describe the reactions. This first order kinetics have a preexponential factor $k_{0,1}$ of 36.13 ${\text{s}}^{-1}$ and an activation energy $E_{0}$ of 43 kJ/mol. The dimensionless energy balance

(8) $$\frac{d\Theta }{d\tau }=\psi \exp\left(\frac{\Theta }{1+\frac{\Theta }{\gamma }}\right)-\Theta$$

is based on the dimensionless temperature Θ, time τ, activation energy γ and the Semenov number ψ

(9) $$\psi =\frac{k_{0,1}e^{-\gamma }m_{TS,0}HHV_{TS}/V_{reactor}}{a_{fw}\alpha_{fw}T_{w}}\gamma$$

(10) $$\Theta =\frac{T-T_{w}}{T_{w}}\gamma$$

(11) $$\tau =\frac{ta_{fw}\alpha_{fw}}{c_{v,f}\frac{m_{f}}{V_{reactor}}}$$

(12) $$\gamma =\frac{E_{0}}{RT_{wall}}$$

The heat transfer coefficient from the fluid to the wall, $\alpha_{fw}$, of 800 $\text{W/}({\text{m}}^{2}\;\text{K})$ was estimated by fitting T(t) trajectories for water/air mixtures to a transient system model (Mangold, 2021). For a wall temperature of 390 °C the critical Semenov number $\psi_{C}$ is 0.4272. Only if $\psi >\psi_{C}$ a thermal runaway is expected. As the amount of feed injected is low, the assumption of no consumption is violated shortly after ignition starts. As a consequence, an ignition peak instead of a thermal runaway is observed. The experimental series with Semenov numbers below $\psi_{C}$, calculated according to Eq. (9), showed no ignition peak. For series with ψ above $\psi_{C}$, an ignition peak was observed. The theoretical limit in TS content for ignition peaks of 13±1 wt% supports the experimental value which was between 11.0±0.2 and 12.9±0.7 wt%.

## 5 Conclusions

• Small amounts of homogenized fecal simulant mixtures can be fed pneumatically at high pressure into an HTO reactor in a reproducible way and without clogging.
• A minimum TOC concentration of 11–13 wt% is needed to produce an ignition peak in both temperature and pressure in a nearly adiabatic batch reactor system.
• Ignition peaks are important safety design parameters. They can be predicted with good accuracy using Semenov’s theory and the kinetics for HTO of feces determined by Hübner et al. (2016).
• An ignition peak is not a prerequisite for reaching high carbon conversions.
• The cyclic batch HTO system FOX-02 reached total carbon conversions from 97 to 99% for a range of feed concentrations (11–15 wt%) at a reactor preheating temperature of 390${}^{\circ }$C, an oxygen-to-carbon ratio ≥1.9, and a minimum reaction time of 600 s.
• TOC values of the process water were in the range of 455–1600 mg/L for a reaction time of 600 s. For a reaction time of 300 s, the TOC values increased to 5600–9900 mg/L. Starting with a feed at 15 wt% TS content, a TOC conversion of at least 99.9% is needed to reach TOC values below 100 mg/L in the process water. To reach this goal, higher reactor temperatures and/or longer reaction times should be explored.
• The FOX-02 system requires 0.7–1.6 $\text{kWh}/{\text{kg}}_{\mathrm{wetf}\mathrm{eed}}$ of external energy to reheat the reactor contents after injecting cold reactants and after rapid discharge. During the oxidation phase at the reactor set-point temperature of 390${}^{\circ }$C, no external heating is needed to sustain the operation due to the heat generation from the exothermic feed conversion.
• Minimizing thermal losses further is critical for attaining a thermally overall self-sustaining HTO operation.

## Declaration of Competing Interest

The authors declare that they have no known competing financial interests or personal relationships that could have appeared to influence the work reported in this paper.

## Data Availability

Data will be made available on request.
